# Investigation on anxiety and depression of different populations in the area with low incidence of New Coronavirus pneumonia and analysis of related factors

**DOI:** 10.12669/pjms.39.2.7136

**Published:** 2023

**Authors:** Yuan Niu, Yincai Liu, Songna Ren

**Affiliations:** 1 Yuan Niu, Xingtai People’s Hospital Office, Hebei Province, P.R. China, 054001; 2 Yincai Liu, Department of Rheumatology, Xingtai People’s Hospital, Hebei Province, P.R. China, 054001; 3 Songna Ren, Xingtai Third Hospital, Hebei Province, P.R. China, 054000

**Keywords:** Anxiety, Depression, Novel coronavirus pneumonia Low incidence area, Influence factor

## Abstract

**Objective:**

To explore the anxiety and depression status and related factors of different populations in the area with low incidence of New Coronavirus pneumonia.

**Methods:**

The anxiety and depression of 106 residents of different ages, different places of residence and different epidemic situations in Xingtai City, Hebei Province, China were assessed from February 18, 2020 to February 20, 2020. The psychological status of different groups was evaluated by self-rating Anxiety Scale (SAS) and self-rating Depression Scale (SDS), and the questionnaire data were analyzed retrospectively. The general data of all residents were collected, and the factors affecting the mental health status of different populations were studied by multiple linear regression with the scores of depression and anxiety as dependent variables.

**Result:**

The SAS and SDS scores of anxiety and depression were (51.79±12.11) and (57.01±11.40) respectively. The positive rates of anxiety and depression were 38.68% and 47.17%, respectively. The results of multiple linear regression analysis showed that marital status, close attention to the daily epidemic progress, and having infected and sick relatives and friends were independent risk factors affecting residents’ SAS score (*P*<0.05). Additionally, marital status, health self-assessment and close attention to the daily epidemic progress every day were independent risk factors affecting residents’ SDS score (*P*<0.05).

**Conclusion:**

Residents in low-incidence areas are prone to anxiety and depression. Marital status, health self-assessment, paying attention to the progress of the epidemic every day and monitoring the disease progress in relatives and friends are independent risk factors contributing to the anxiety and depression of the residents. Corresponding protective measures should be taken to improve the local epidemic prevention and control level.

## INTRODUCTION

First cases of novel coronavirus pneumonia were found in Wuhan city of Hubei province in late 2019. In January 2020, World Health Organization (WHO) named New Coronavirus pneumonia (later referred to as “coronavirus disease, or COVID-19”) as class B infectious diseases. It was controlled according to the infectious diseases of class A in January 2020.^1^-^3^ Despite control measures, novel coronavirus pneumonia rapidly spread throughout the country and globally. Due to its strong infection potential, as well as wide range and rapid spread of the disease, WHO declared it as the epidemic on January 30, 2020.^4^,^5^ Clinical manifestations of COVID-19 in infected patients are weakness, fever, cough, runny nose, diarrhea etc. Patients with severe progression of the disease may develop multiple life-threatening organ dysfunctions, such as acute respiratory distress syndrome, septic shock and respiratory failure.^6^-^8^

At present, there is no pharmacological treatment for the novel coronavirus pneumonia, and if not treated promptly, it may lead to death. The pathogen of COVID-19 has a high homology with SARS coronavirus and Middle East respiratory syndrome coronavirus, all of which were considered major public health emergencies.^9^,^10^ Such incidents are likely to have a profound psychological and mental impact on general population. Due to the uncontrollable and uncertain nature of the epidemic, it can cause anxiety, depression, panic, tension, and other negative psychological effects.^11^,^12^ While many aspects of the novel coronavirus pneumonia were investigated in the past few years, the research on anxiety and depression problems of residents and different populations in the low incidence area is scarce. And the mental health problems of residents in different regions, cultures and epidemic prevention policies will also be different. The main goal of this study was to analyze and compare psychological status of residents in Xingtai, Hebei province, to explore the influencing factors of anxiety and depression, and to provide scientific basis for effective regulations, aimed to improve psychological status and mental health of residents in low incidence areas.

## METHODS

The survey results of mental health status of 106 residents of different ages, different places of residence and different epidemic diagnosis in Xingtai City, Hebei Province from February 18, 2020 to February 20, 2020 were analyzed retrospectively.

### Inclusion criteria:

- Living in Xingtai City, Hebei Province;
- The questionnaire and relevant data are complete;
- Age ≥ 18 years;
- Able to complete questionnaire survey and data filling independently;
- Meet the ethical review standards of the Helsinki declaration.

### Exclusion criteria:

- Missing data and questionnaire;
- Cognitive impairment, abnormal audio-visual and expression abilities;
- Mental and brain diseases;
- Diagnosis of COVID-19.

General information questionnaire, prepared by survey organizers and researchers, included gender, age, place of residence, diagnosis of relatives and friends, marital status, occupation type, education level, average monthly income, self-assessment of health, understanding of epidemic-related knowledge, daily attention to epidemic progress, regional epidemic prevention and control, etc. Self-rating Anxiety Scale (SAS), developed by Zung^13^ was used. The scale contains 20 items. Likert 4-level scoring method was adopted, where responses are given on a 4-point scale which range from one (none, or a little of the time) to four (most, or all of the time). Higher scores correlate with more severe anxiety.

Scores below 50 points indicate lack of anxiety, between 50 and 59 points indicate mild anxiety, between 60 and 69 points represent moderate anxiety, and above 69 points represent severe anxiety. The scale has good reliability and validity, with Cronbach’s α at 0.884.^3^ Self rating Depression Scale (SDS) was developed by Zung.^14^ It has 20 items and adopts Likert grade Four scoring method that rates the four common characteristics of depression: the pervasive effect, the physiological equivalents, other disturbances, and psychomotor activities. Each question is scored on a scale of 1-4 (a little of the time, some of the time, good part of the time, most of the time). The full score is 80 points, of which scores of 53~60 points represent mild depression, 61~70 points represent moderate depression, 71 ~ 80 points represent severe depression. The scale has good reliability and validity, and the Cronbach’s α coefficient of 0.839.

Surveys included in this study were conducted through the “questionnaire star” platform questionnaire or paper questionnaire. To ensure the quality of the survey, only one survey was conducted for each IP address, and the data with an answer time of more than three minutes was considered valid. The collection, storage, use, transmission and processing of interviewees’ information was in the charge of special personnel, and a strict confidentiality system was implemented. Before the survey, all participants of the research group were familiarized with the contents of the questionnaire and trained on the use of the questionnaire star platform. During the training, relevant course training documents were distributed to the participants of the research group, and communication links were established among the participants. A total of 110 residents in Xingtai, Hebei Province participated in the survey, including residents of different ages, different places of residence and confirmed infection of relatives and friends. A total of 110 questionnaires were collected, of them 106 questionnaires met the inclusion criteria, with the effective questionnaire recovery rate of 96.36%. All questionnaires were strictly reviewed by relevant personnel and were included in the study after confirming that there were no omissions or mistakes. This study was approved by the Medical Ethics Committee of Xingtai People’s Hospital (Approval No.: LL2021.39; Date: May 24, 2022).

### Statistical Analysis:

SPSS 22.0 statistical software was used for data analysis. The measurement data were expressed in (), t-test was performed for pairwise comparison, and ANOVA variance was used for multi group comparison. General data of all residents were collected, and the factors affecting the mental health status of different populations were studied by multiple linear regression with the scores of depression and anxiety as dependent variables. P < 0.05 was considered significant.

## RESULTS

Xingtai city of Hebei province is a low-incidence area of COVID-19. The anxiety and depression scores of 106 residents in the study were (51.79±12.11) and (57.01±11.40) respectively. Among 106 residents, the positive rate of anxiety symptoms was 38.68% (41 / 106), including 20 cases of mild depression, 16 cases of moderate depression and five cases of severe depression. The positive rate of depressive symptoms was 47.17% (50 / 106), including 31 cases of mild depression, 13 cases of moderate depression and six- cases of severe depression. There was no significant difference in SAS and SDS scores among different age groups (*P*>0.05). Table-I There was no significant difference in SAS and SDS scores between groups in different places of residence (*P*>0.05). Table-II

**Table-I:** Anxiety and depression status and age.

Group	n	SAS score	SDS score
18~30 years	33	49.32±12.58	52.64±11.25
30~50 years	44	46.13±13.22	49.68±12.50
>50 years	29	44.85±10.26	47.79±10.84
*F*		1.126	1.375
*P*		0.328	0.257

**Table-II:** Anxiety and depression of people in different places of residence

Group	n	SAS score	SDS score
City proper	55	47.30±12.97	50.59±11.63
Town	31	48.11±12.95	51.69±12.05
Rural	30	47.50±11.76	51.20±11.17
*F*		0.041	0.092
*P*		0.960	0.912

The SAS and SDS scores of participants with coronavirus-infected relatives and friends were significantly higher than those of who had no infected relatives and friends (*P*<0.05). Table-III There were statistically significant differences in SAS and SDS scores among people with different marital status, educational level, health self-assessment, knowledge about the epidemic situation and paying close attention to the daily reports of the epidemic progression (*P*<0.05). There were no statistically significant differences in gender, occupation type, monthly average income and regional epidemic prevention and control ability (*P*>0.05). Table-IV

**Table-III:** Anxiety and depression and infection among friends and family

Group	n	SAS score	SDS score
Infected relatives and friends	32	52.78±12.97	55.28±11.31
Uninfected relatives and friends	74	46.32±12.31	49.97±11.46
*t*		2.441	2.199
*P*		0.016	0.030

**Table-IV:** Univariate analysis of SAS and SDS scores

Item	n	SAS score	t/F	P	SDS score	t/F	P
Sex			0.032	0.974		0.022	0.982
Male	55	47.89±12.77			50.49±11.52		
Female	51	47.81±12.81			50.54±11.48		
Marital status			3.298	0.041		4.524	0.013
Unmarried	39	47.13±12.95			51.19±11.12		
Married	51	47.26±12.87			50.14±11.87		
Widowhood or divorce	16	56.13±12.26			59.90±11.32		
Occupation type			0.268	0.766		0.443	0.643
Manual work	42	48.00±12.33			51.45±11.83		
Mental work	40	46.25±13.01			49.40±11.52		
Unemployed or retired	24	48.26±12.63			51.79±11.36		
Education level			3.346	0.039		4.416	0.014
Junior high school and below	11	54.83±10.97			56.26±10.97		
High school or college	52	48.65±12.90			52.41±11.56		
Bachelor degree or above	43	44.40±12.74			46.71±11.60		
Average monthly income (Ten thousand yuan)			1.402	0.251		2.400	0.096
<0.5	43	49.98±12.50			54.01±11.32		
0.5~1.0	53	48.05±13.06			50.98±11.17		
>1.0	10	42.60±10.97			45.82±10.87		
Health self-assessment			5.956	0.004		7.385	0.001
Excellent	61	45.77±12.50			49.24±11.30		
Regular	36	52.50±11.41			55.61±10.30		
Poor	9	57.44±10.76			61.70±10.52		
Knowledge of epidemic situation			3.457	0.035		3.419	0.037
Do not understand	10	56.73±11.88			57.39±10.15		
Commonly	52	48.01±12.10			53.72±11.13		
Understand	44	45.39±12.74			48.88±11.71		
Pay attention to the epidemic every day			2.283	0.024		3.076	0.003
Yes	85	46.91±12.55			50.23±11.42		
No	21	53.90±12.62			58.70±10.78		
Regional epidemic prevention and control			1.975	0.144		1.916	0.152
Strict	70	46.39±12.40			49.90±11.41		
Commonly	28	50.31±12.88			53.87±11.43		
Loosely	8	54.03±12.69			56.09±12.21		

Multiple linear regression analysis showed that marital status, paying attention to the epidemic progress every day and having coronavirus-infected relatives and friends were independent risk factors affecting residents’ SAS scores; Marital status, self-assessment of health and paying attention to the progress of epidemic situation every day were independent risk factors affecting residents’ SDS score (*P*<0.05). Table-V.

**Table-V:** Multivariate analysis of SAS scores and SDS scores

SAS scores					SDS scores				

Item	Reference group	Regression coefficient	Partial regression coefficient	t	P	Regression coefficient	Partial regression coefficient	t	P
Constant	-	0.369	-	3.637	0.000	0.232	-	1.668	0.098
Marital status	Unmarried	0.564	0.796	8.290	0.000	0.286	0.393	3.068	0.003
Education level	Junior high school and below	0.001	0.001	0.006	0.995	0.002	0.002	0.008	0.994
Health self-assessment	Excellent	-0.03	-0.04	-0.460	0.646	0.217	0.282	2.438	0.017
Knowledge of epidemic situation	Do not understand	0.128	0.168	0.868	0.387	0.210	0.268	1.039	0.301
Paying attention to the epidemic every day	Yes	-0.379	-0.310	-4.757	0.000	-0.248	-0.198	-2.274	0.025
Diagnosis of epidemic situation	Infected relatives and friends	0.258	0.243	3.403	0.001	0.156	0.143	1.504	0.136

***Note:*** SAS scores R^2^ = 0.848, adjusted R^2^ = 0.839, f = 92.377, P < 0.001; SDS scores R^2^ = 0.730, adjusted R^2^ = 0.713, f = 44.526, P < 0.001.

## DISCUSSION

The results of our study show that among 106 residents, included in the study, the positive rate of anxiety symptoms was 38.68% and the positive rate of depressive symptoms was 47.17%. These results indicate that even in a low-risk area, many residents still have different degrees of mental health problems. Most people are not well-equipped to cope with such emergencies as the recent COVID-16 epidemic. This can result in stress, anxiety, depression, fear, and other negative emotions that are not conducive to mental health, can lead to abnormal and irrational behavior and have an overall serious negative impact on general population.^15^,^16^

Salari N et al^17^ conducted a meta analysis and found the prevalence of stress in five studies with a total sample size of 9074 is obtained as 29.6% (95% confidence limit: 24.3-35.4), the prevalence of anxiety in 17 studies with a sample size of 63,439 as 31.9% (95% confidence interval: 27.5-36.7), and the prevalence of depression in 14 studies with a sample size of 44,531 people as 33.7% (95% confidence interval: 27.5-40.6). This time, the subjects were divided into three age groups for comparison, SAS and SDS scores were not statistically significant, but Varma P et al^18^ studied that younger people were more likely to have anxiety and depression, possibly due to different regions. We showed that marital status, self-assessment of health, paying close attention to the progress of epidemic situation every day and having relatives and friends that contracted the disease are independent risk factors affecting residents’ SAS and SDS scores. We may speculate, that widowed or divorced residents mostly live alone or with their families and children.

When an emergency occurs, these people may exhibit lower psychological tolerance and lack of confidence in coping with the epidemic, are more worried about their health and the situation with their families.^10^ People with underlying medical conditions are considered at higher risk of contracting novel coronavirus pneumonia and of possible complications of the disease.^19^ Therefore, people that self-assess their condition as poor may be more prone to depression, serious psychological problems and loss of confidence in their ability to fight the epidemic.^17^ The SAS and SDS scores of residents who pay close attention to the progress of the epidemic on a daily basis are lower.

This may be due to the fact that residents who actively understand and process the information related to the epidemic have a higher level of knowledge of the epidemic and have a better cognitive level.^16^,^17^ They are less likely to listen to false information, which may affect their judgment. Residents who have infected relatives and friends have higher scores of anxiety and depression, due to the increased sense of uncertainty and threat caused by the disease in their close environment.^20^ The results of our study further strengthen the notion that targeted measures, aimed at improving the control of the disease, will be highly beneficial for the mental health of residents. Special attention should be given to people with self-assessed poor health, people who fail to pay close attention to the progression of the epidemic, and people who have infected and sick relatives and friends, to focus on improving their psychological state, reduce levels of stress, anxiety and depression.^19^,^20^ It is hoped that this study will help prevent the harmful effects of the wave of COVID-19 or similar collective stress events in the future, and provide a basis for psychological counseling and intervention strategies for different populations in low incidence areas.

### Limitations:

Sample size of the survey is limited. Additionally, this is a single- center research. Further multi-center studies with larger sample sizes are needed for more convincing and representative results.

## CONCLUSION

Our study shows that the marital status, self-evaluation of health, infected friends and relatives and ability to follow the progression of the COVID-19 epidemic daily are independent risk factors contributing to the anxiety and depression of the residents in low incidence areas. Effective epidemic prevention and psychological counseling measures should be taken according to the personal situation of residents to protect their physical and mental health and improve the overall effect of local epidemic prevention and control.

### Authors’ contributions:

**YN** conceived and designed the study.

**YL** and **SR** collected the data and performed the analysis.

**YN** was involved in the writing of the manuscript and is responsible for the integrity of the study.

All authors have read and approved the final manuscript.
